# Weaponising microbes for peace

**DOI:** 10.1111/1751-7915.14224

**Published:** 2023-03-07

**Authors:** Shailly Anand, John E. Hallsworth, James Timmis, Willy Verstraete, Arturo Casadevall, Juan Luis Ramos, Utkarsh Sood, Roshan Kumar, Princy Hira, Charu Dogra Rawat, Abhilash Kumar, Sukanya Lal, Rup Lal, Kenneth Timmis

**Affiliations:** ^1^ Department of Zoology Deen Dayal Upadhyaya College, University of Delhi Delhi India; ^2^ Institute for Global Food Security, School of Biological Sciences Queen's University Belfast Belfast UK; ^3^ Athena Institute for Research on Innovation and Communication in Health and Life Sciences Vrije Universiteit Amsterdam Amsterdam The Netherlands; ^4^ Center for Microbial Ecology and Technology (CMET) Ghent University Ghent Belgium; ^5^ Department of Medicine Johns Hopkins School of Public Health and School of Medicine Baltimore Maryland USA; ^6^ Estación Experimental de Zaidin, CSIC Granada Spain; ^7^ Department of Zoology Kirori Mal College, University of Delhi Delhi India; ^8^ Post‐Graduate Department of Zoology Magadh University Bodh Gaya Bihar India; ^9^ Department of Zoology Maitreyi College, University of Delhi New Delhi India; ^10^ Department of Zoology Ramjas College, University of Delhi Delhi India; ^11^ PhiXgen Pvt. Ltd Gurugram, Gurgaon Haryana India; ^12^ Acharya Narendra Dev College, University of Delhi Govindpuri, Kalkaji, New Delhi India; ^13^ Institute of Microbiology, Technical University Braunschweig Braunschweig Germany

## Abstract

There is much human disadvantage and unmet need in the world, including deficits in basic resources and services considered to be human rights, such as drinking water, sanitation and hygiene, healthy nutrition, access to basic healthcare, and a clean environment. Furthermore, there are substantive asymmetries in the distribution of key resources among peoples. These deficits and asymmetries can lead to local and regional crises among peoples competing for limited resources, which, in turn, can become sources of discontent and conflict. Such conflicts have the potential to escalate into regional wars and even lead to global instability. Ergo: in addition to moral and ethical imperatives to level up, to ensure that all peoples have basic resources and services essential for healthy living and to reduce inequalities, all nations have a self‐interest to pursue with determination all available avenues to promote peace through reducing sources of conflicts in the world. Microorganisms and pertinent microbial technologies have unique and exceptional abilities to provide, or contribute to the provision of, basic resources and services that are lacking in many parts of the world, and thereby address key deficits that might constitute sources of conflict. However, the deployment of such technologies to this end is seriously underexploited. Here, we highlight some of the key available and emerging technologies that demand greater consideration and exploitation in endeavours to eliminate unnecessary deprivations, enable healthy lives of all and remove preventable grounds for competition over limited resources that can escalate into conflicts in the world. We exhort central actors: microbiologists, funding agencies and philanthropic organisations, politicians worldwide and international governmental and non‐governmental organisations, to engage – in full partnership – with all relevant stakeholders, to ‘weaponise’ microbes and microbial technologies to fight resource deficits and asymmetries, in particular among the most vulnerable populations, and thereby create humanitarian conditions more conducive to harmony and peace.

## Graphical abstract

Deficits and asymmetries in basic resources and services considered to be human rights, such as a healthy diet, drinking water, sanitation and hygiene, healthcare, and a clean environment, can create discontent that may lead to conflicts, some of which have the potential to escalate into regional wars that in turn may cause global instability. Here, we highlight some of the available and emerging microbial technologies that demand greater consideration and exploitation in endeavours to eliminate unnecessary deprivations, enable healthy lives of all, and remove preventable grounds for conflicts in the world. Such technologies are preordained as some of the key mediators of social equity and peace among and between peoples.

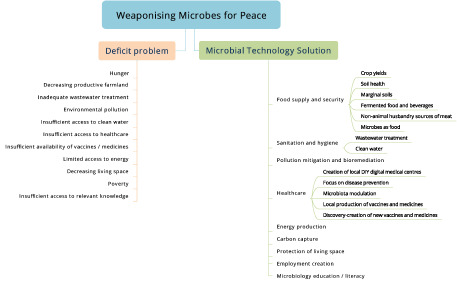

**Glossary**
*Agrobiochemicals*
: Agrochemicals of biological origin, such as metabolites from microbes or plants that are used as pesticides on crop plants (including pyrethrins and many photosensitisers; Braga et al., 2022), microbial enzymes added to cattle feed, etc.
*Agrobiologics*
: Organisms (typically microbes that are physiologically active or dormant‐yet‐viable) that are cultured and used to enhance agricultural productivity, including biopesticides/biocontrol agents; microbes used as plant, seed or soil inoculants to promote plant health or plant growth (biostimulants such as mycorrhizal fungi and nitrogen‐fixing bacteria) or improve soil structure and/or soil fertility
Agrochemicals: Substances used in agriculture, such as chemical pesticides, fertilisers, plant‐growth substances and animal hormones.
Biocontrol agents: Biological control agents are microorganisms used in agriculture and horticulture to control pathogens and pests including [bacteria applied to leaves], entomopathogenic fungi, and [viruses?]. The biocontrol agents are a subset of ‘*Agrobiologics*’ and the term ‘biocontrol agents’ is synonymous with ‘Biopesticides’ and ‘Microbial pesticides’.
Biopesticides: Another term for ‘Biocontrol agents’.
Bioremediation: An umbrella term for processes by which microorganisms or plants are used to de‐contaminate polluted environments or materials. Examples include the use of bacteria to break down xenobiotics such as pesticide residues; the use of oil‐degrading microbes to catabolise crude oil in seawater, groundwater or soils; the use of fungi or plants to take up and accumulate heavy metals (prior to their biomass being destroyed/processed); and the use of microbes to degrade plastic contamination.
Bushmeat: The meat of wild land animals (especially mammals but also some amphibians, reptiles, and birds) that are consumed in humid tropical regions of the world. Bushmeat is a subset of ‘Wild meat’.
Clean water: Is water—such as the freshwater in aquifers, rivers, lakes, reservoirs and tapwater—that is not significantly contaminated (e.g. by xenobiotic pollutants, radioactive chemicals, heavy metals, sewerage or other microbes) and is considered clean enough to drink, according to criteria such as those of the World Health Organization (*Guidelines for Drinking‐Water Quality: Fourth Edition Incorporating the First and Second Addenda*. World Health Organization (9789240045064‐eng.pdf)).
Cold chain: A temperature‐controlled supply chain/network whereby foods, vaccines, or other perishable products are transported, stored and distributed under chilled conditions to extend longevity, in particular, to enable the use of such products in warm climates.
Conflict‐driven resource deficits: The conflict‐driven development of deficits in basic resources and services essential to healthy lives.
Conflicts: Conflicts—whether political, economic and/or physical (including war)—between individuals, societies, nations and/or groups of nations.
Deficit: The lack of or inadequate access to a basic resource or service essential for healthy living.
Disease‐suppressive soils: Soils that temporarily or more persistently contain plant pathogens that cause less damage than they ordinarily would in normal soils: their pathogenic activities are repressed by the soil microbiota.
Earth's biosphere: The domain on Earth populated by organisms (whether physiologically active or dormant) including the subsurface, oceans, land surface and atmosphere (Hallsworth et al., 2021; Šantl‐Temkiv et al., 2022).
Germophobia: An irrational fear of microorganisms based on an assumption they are pathogenic. Typically accompanied with an obsession with personal hygiene and cleanliness of a person's environment (e.g. disinfecting/sterilising artefacts and surfaces).
High‐income country/ies (HICs): Defined by the World Bank as a nation with a gross national income per capita of US$12,696 or more in 2022, calculated using the Atlas method (http://data.worldbank.org/about/country‐and‐lending‐groups#High_income).
Hunter–gatherers: Peoples or individuals now or in the past who subsisted by hunting wild animals and foraging wild plants for food and other purposes (rather than rearing livestock or growing plants for food and other purposes).
Levelling‐up: The process of reducing resource asymmetries between peoples.
Low‐to‐medium income country/ies (LMICs): low‐income countries are defined by the World Bank as having a gross national income per capita of US$1,045 or less in 2022. Medium income countries are defined as having a gross national income per capita between US$ 12,695 and US$1,046 in 2022 ( http://data.worldbank.org/about/country‐and‐lending‐groups#High_income)
Indigenous peoples: ‘culturally distinct ethnic groups whose members are directly descended from the earliest known inhabitants of a particular geographic region and, to some extent, maintain the language and culture of those original peoples’ (https://en.wikipedia.org/wiki/Indigenous_peoples).
Marginal soils: Soils considered to be uneconomic to farm because the effort and/or financial investment needed to farm them is typically greater than the yield or financial worth of the crop obtained. Soils may be unproductive due to desertification or low water‐holding capacity, waterlogging, a lack of nutrients or low cation‐exchange capacity, pollution, low content of organic matter and humic acids or other reasons.
Marginalised community: Groups of people excluded from or confined to the edge of society
Microbial desalination cells: A microbial technology involving electroactive bacteria that mediates both desalination and degradation of organic wastes, and thereby generates freshwater with little energy input (in contrast to the established reverse osmosis technology)
Microbial foods: Foods that consists of microbial biomass, such as mushrooms or other fungi (including mycoprotein products such as Quorn), and increasingly Cyanobacteria like *Spirulina* (now *Arthrospira*)
(Neo‐)adjuvant/adjunct therapies: (Neo)adjuvant or adjunct therapy is given as a complement to a primary course of treatment, for example various types of biotics or faecal microbiota transplant. Neoadjuvant therapy is given *a priori* to, for example modulate gut microbiota for better facilitation of, for instance, chemotherapy. Adjuvant or adjunct therapy might be given in parallel or after the primary course of treatment.
Pareto distribution: The Pareto Principle or 80:20 distribution describes the situation where 80% of a resource is exploited by 20% of the users https://en.wikipedia.org/wiki/Pareto_principle. The Principle was originally described by Pareto in the context of land ownership but has since been shown to apply in many situations.
Peace: Harmony between individuals/peoples/nations, the converse of which is conflict.
Phytoremediation: ‘technologies use living plants to clean up soil, air and water contaminated with hazardous contaminants…’ the use of green plants and the associated microorganisms, along with proper soil amendments and agronomic techniques to either contain, remove or render toxic environmental contaminants harmless (Das, 2018)“ (https://en.wikipedia.org/wiki/Phytoremediation).
Plant growth‐promoting inocula: Microorganisms added to seeds, plants or soils that enhance plant growth by aiding nutrient and/or water uptake, converting gaseous nitrogen to nitrogenous solutes that can be used as nutrients by plants, regulation of plant‐growth substances, and increasing stress tolerance of plants. All these services are also afforded to wild plants by microorganisms in Nature.
Plant:microbe partnerships: Plants host populations of diverse microbes on their surfaces—the epiphytes—and inside their organs and cells—the endophytes, which collectively are called the microbiome. Both plant and microbes provide each other with key resources and services. In addition, some microbes are more loosely associated with plant roots: this region of plant root:microbe interaction is called the rhizosphere. While these mutually beneficial interactions are key for plant (and soil) health, they may not be optimal for agricultural or biotechnological endeavours by humans. In this case, it may be possible to modify the composition of the microbiome or rhizosphere to create new plant:microbe partnerships more effective for the application at hand.
Pollution mitigation: Use of microbes (and plants) to clean up contaminated sites.
Polydeficits: The existence of multiple, often interacting and reinforcing, deficits experienced mostly by disadvantaged peoples
Pro‐/pre‐/syn‐/post‐/pharmacobiotics: Probiotics are ‘live microorganisms which when administered in adequate amounts confer a health benefit on the host’. https://www.nature.com/articles/nrgastro.2014.66. A prebiotic is ‘a substrate that is selectively utilised by host microorganisms conferring a health benefit’. https://www.nature.com/articles/nrgastro.2017.75. A synbiotic is ‘a mixture comprising live microorganisms and substrate(s) selectively utilised by host microorganisms that confers a health benefit on the host’. https://www.nature.com/articles/s41575‐020‐0344‐2. A postbiotic is a ‘preparation of inanimate microorganisms and/or their components that confers a health benefit on the host’. https://www.nature.com/articles/s41575‐021‐00440‐6. Pharmabiotics are ‘bacterial cells of human origin, or their products, with a proven pharmacological role in health or disease’. https://www.ncbi.nlm.nih.gov/pmc/articles/PMC3026447/
Road kill: Animals accidentally killed on roads by vehicular traffic.
Societal benefits: Benefits to human society(ies) that arise from microorganisms/microbial technologies (including those technologies associated with *agrobiologics* and bioremediation).
Societally‐relevant microbiology: Aspects of the discipline microbiology, especially those we perceive as negative, such as infectious diseases, and those we consider positive that are provided by technologies based on microbial activities, which are particularly relevant to society, sustainability, and the management of a number of current crises.
Soil fertility: The ability of soil(s) to support plant growth/plant productivity, most often‐used in the context of crop‐plant yields. Whereas crop yields may vary because some plants have requirements/preferences for specific types of soil, the concept of soil fertility in general assumes a weed‐like phenotype where the crop grows best in an open habitat without the need for specific or extreme plant‐growth conditions (Anderson, 1952). Soil fertility is reduced if there are low soil nutrients or poor soil structure. Fertile agricultural soils typically have good drainage and good water‐holding capacity.
Soil health: ‘a state of a soil meeting its range of ecosystem functions as appropriate to its environment. In more colloquial terms, the health of soil arises from favourable interactions of all soil components (living and non‐living) that belong together, as in microbiota, plants and animals. It is possible that a soil can be healthy in terms of eco‐system functioning but not necessarily serve crop production or human nutrition directly, hence the scientific debate on terms and measurements’ (https://en.wikipedia.org/wiki/Soil_health).
Soil inoculants: Microbes (whether physiologically active or dormant propagules) added to soils for various purposes: usually, to improve soil health and/or plant health. Examples include nitrogen‐fixing bacteria that are symbionts of leguminous plants, microbes used as biofertilisers and microbes from disease‐suppressive soils. Soil inoculants are a subset of ‘*Agrobiologics*’ and ‘Biofertilisers’.
Soil nutrients: Chemical elements in soils that are available for use as nutrients by microbes and plants, especially nitrogen, phosphorus, potassium, calcium, magnesium and sulfur. The nutrient status of soils can depend on its cation‐exchange capacity because changed components of soil (such as clay particles and humic acids) can withhold and then release ions into the soil solution.
Soil structure: according to The Royal Society ‘Soil structure refers to the arrangement of solids and pore spaces within soil. Solids, formed from organic compounds and mineral ions clump together to form aggregates. The gaps between these aggregates are the pore spaces. For soil used in agriculture, a “well‐structured soil” will have a continuous network of pore spaces to allow drainage of water, free movement of air and unrestricted growth of roots’ (https://royalsociety.org/‐/media/policy/projects/soil‐structures/soil‐structure‐evidence‐synthesis‐report.pdf).
Stress [in plants]: A physiological condition where growth rate, physiology, and/or health are adversely compromised. This usually triggers stress responses or adaptations in the plant and often requires upregulation of energy generation. Stresses can be induced by temperature extremes, freezing, dehydration/desiccation, salt, extremes of pH, etc.
Sustainable development: ‘Sustainable development is an organising principle for meeting human development goals while also sustaining the ability of natural systems to provide the natural resources and ecosystem services on which the economy and society depend…defined in the 1987 Brundtland Report as “Development that meets the needs of the present generation without compromising the ability of future generations to meet their own needs” (https://sustainabledevelopment.un.org/content/documents/5987our‐common‐future.pdf). As the concept of sustainable development developed, it has shifted its focus more towards the economic development, social development and environmental protection for future generations’ (https://en.wikipedia.org/wiki/Sustainable_development).
Sustainable Development Goals (SDGs): According to the United Nations ‘The 2030 Agenda for Sustainable Development, adopted by all United Nations Member States in 2015, provides a shared blueprint for peace and prosperity for people and the planet, now and into the future. At its heart are the 17 Sustainable Development Goals (SDGs), which are an urgent call for action by all countries—developed and developing—in a global partnership. They recognize that ending poverty and other deprivations must go hand‐in‐hand with strategies that improve health and education, reduce inequality and spur economic growth—all while tackling climate change and working to preserve our oceans and forests’. (https://sustainabledevelopment.un.org/post2015/transformingourworld)
WASH: Term coined by the World Health Organization to encompass drinking water, sanitation and hygiene (https://www.who.int/publications/i/item/9789240065031)
Weaponising: This term is used figuratively to express the harnessing of microbes and microbial activities in technical processes implemented to eliminate deficits and asymmetries in essential resources and services within and between human societies/nations, with the aim of reducing causes of conflicts and hence promoting peace.
Wet markets: Markets selling perishable foods usually including meat and fish. Often with multiple stalls/stall holders and sometimes in an informal setting in a large market‐hall or outdoors.
Wild meat: The meat of wild animals (especially land animals and birds) that is eaten by humans in all/any part(s) of the world.

## CONFLICTS, PEACE AND MICROBES

Peace, and its absence – political division, aggression, invasion and warfare – have preoccupied humanity since *Homo sapiens* diverged from the other apes. Conflicts often result from interpersonal or otherwise local or regional tensions, but can readily become international as a result of geopolitical activities to gain/maintain power and influence, and to secure or dominate supplies of strategically important natural resources, often through proxy wars, with nations supporting one or other of the combatants, as exemplified in conflicts in the Syria (2011 onwards), Yemen (2014 onwards), Ethiopia (2020–2022) and Ukraine (2014 and 2022 onwards). The current conflict in Ukraine is a prime example of how a regional conflict can precipitate global consequences, in this case in provoking crises of global food security and energy supplies, thereby undermining national economies worldwide. And, of course, the risk of a conflict leading to a nuclear conflagration that may destroy both human society and the Earth's biosphere as we know them can never be excluded (e.g. see Timmis & Hallsworth, 2022).

There exist many causes of human‐driven conflicts, including distrust, societal disparities, anomie (the disconnect between individual beliefs/values and social norms) accompanied by the search for simplistic explanations of complex phenomena (conspiracy theories), othering, adverse personal characteristics of powerful individuals, or extremism (be it rooted in dogma, perceived incompatibility with or inferiority of other belief systems, racism and other kinds of xenophobia, or other equally divisive worldviews). But an arguably more desperate (and some might consider potentially more justifiable and rationalisable) cause/motive is serious regional deficits in fundamental resources needed for the members of those societies to live healthy and dignified lives and that might generally be considered to constitute basic human rights.

Moreover, there are often substantial asymmetries between communities, regions and nations in the availability and supply of such resources. Such asymmetries are inconsistent with sustainable development of humanity, and their elimination lies at the heart of the Sustainable Development Goals (SDGs) formulated by the United Nations (https://sdgs.un.org/2030agenda), such as SDG 10: *Reduce inequality within and among countries*. The reasons for the asymmetries can be either natural, a consequence of geography, climate, etc., or caused and perpetuated by humans, such as the (post‐)colonial redistribution of resources from the global south to the global north.

Such deficits and asymmetries, and efforts to locally increase supplies to adequate levels, create competition between neighbours for limited supplies and, in turn, sometimes the potential for conflict. And, importantly, they may also be weaponised by unscrupulous leaders to demonise others and propagate self‐serving conflicts.

There is an urgent need for proactive measures to reduce the causes of local conflicts. Quite apart from the humanitarian imperative to reduce the human suffering caused by deficits in basic resources, and conflicts engendered by them, the international community has a vital self‐interest in diminishing causes of conflict, and promoting social equity, harmony and peace among peoples. Worldwide elimination of deficits in basic resources must become a pivotal element of strategic policy to secure peace among nations.

‘Microbes’ and ‘peace’ are not terms that are usually associated with one another. In fact, to most people, it might well appear that we are constantly at war with microbes. We talk about *killing* bacteria, *fighting* infection, antimicrobial *resistance*, *battles* on mucosal surfaces, *biocides*, *disinfectants*, *pesticides* and other weapons against pathogens. Personal care products and household cleaning products, kitchen counter tops, paints, etc., often contain antimicrobial substances, as do foods (such as salt, sugar, ethanol, nitrites and other preservatives). However, most microbes and, most importantly, their associations with one another, with other organisms and with the physical components of the biosphere, provide vital ecosystem services essential to our well‐being and that of the entire biosphere. Indeed, such microbial associations, particularly the manner in which microbes cooperatively interact to maximise exploitation of available resources (e.g. see Pelz et al., 1999; Venkataram et al., 2023), can provide guidance on how societies might behave more cooperatively and harmoniously, in particular when pursuing identical goals. Here we argue that technologies based on microbes and microbial societies have unprecedented potential to serve as facilitators of peace in the world.

## WHAT ARE THE MAJOR PROBLEM DEFICITS TO WHICH MICROBIAL TECHNOLOGY CAN CONTRIBUTE?

Clearly there are many types of deficits and asymmetries, even among High‐income countries (HICs), so the following list is neither comprehensive nor necessarily balanced, but is meant to exemplify some of the major problems:

- hunger: approximately 10% of the world's population suffer from hunger (www.fao.org/state‐of‐food‐security‐nutrition/2021/en; SDG 2). This statistic needs to be appreciated in the context of another, namely that one third of all food produced in the world is wasted (https://www.stopfoodlosswaste.org/issue),
- decreasing productive farmland, which in some cases is the direct cause of hunger. The current episode of human‐driven global climate change is causing reduced rainfall in some regions, leading to increasing desertification and loss of productive farmland, and soil losses due to wind erosion (Burrell et al., 2020), and increased rainfall in other regions, leading to flooding and loss of fertile soil by water erosion. The decreasing productive farmland, in turn, leads to falling agricultural productivity (SDG 15) and food security,
- inadequate wastewater treatment: approximately half of the world's population lack access to safe sanitation facilities (https://www.unicef.org/stories/state‐worlds‐sanitation), 6% practice open‐air defecation, and each year 1% die as a result of inadequate water, sanitation and hygiene (https://www.who.int/news‐room/fact‐sheets/detail/sanitation; SDG 6),
- lack of clean water: one in three have no or inadequate access to safe drinking water (https://www.who.int/news‐room/fact‐sheets/detail/sanitation; SDG 6),
- insufficient basic health coverage and access to healthcare: almost 40% of the world's population does not have access to basic health services and effective medicines (https://www.who.int/news/item/13‐12‐2017‐world‐bank‐and‐who‐half‐the‐world‐lacks‐access‐to‐essential‐health‐services‐100‐million‐still‐pushed‐into‐extreme‐poverty‐because‐of‐health‐expenses; SDG 3).
- limited living space: there is an increasing displacement of peoples from rural areas to cities, as well as to coastal regions. This trend will increase dramatically as climate change and rising sea levels, caused by global warming, squeeze habitable zones and lead to further human migrations, thereby increasing competition for living space in other parts of the world (SDGs 10, 11, 13). Moreover, poverty, hunger, disease – all of which may be exacerbated by climate change (e.g. https://www.usip.org/publications/2022/09/how‐climate‐change‐catalyzes‐more‐migration‐central‐america) are also major factors promoting mass migrations and overcrowding in destination lands. And, of course, persecution, victimisation, discrimination, maltreatment and war itself have displaced large numbers of peoples, many of which are confined long term to refugee camps that are often horrifically overcrowded and lack many forms of basic resources.
- deforestation, that reduces biodiversity ecosystem services, including carbon sequestration, water retention, soil stability and fertility. It also favours an increase in the incidence and spread of zoonotic infections through increased contacts of humans with wildlife as a result of humans entering wildlife habitats (Wolfe et al., 2004), and the displacement of wildlife from their natural habitats into living space that humans consider their own (SDG 15),
- limited access to, and insufficient resources and technology to generate, energy (SDG 7),
- pollution of soils, waters and air, mostly but not always as a result of industrial activity (often as a result of extracting raw materials for export), which not only impacts human health but also reduces biodiversity and the services it provides,
- poverty: 10% of the world's population live in extreme poverty (SDG 1) with little access to decent work and income (SDG 8),
- limited access to education (SDG4), in the context of this discussion, to microbiology education, expertise and know‐how related to well‐being, the production of food, clean water, energy, sustainable practices, environmental protection and conservation of ecosystem services.

Crucially, one basic resource deficit is usually accompanied by others, creating a *polydeficit* calamity, since resource deficits are by nature interdependent and to some extent mutually reinforcing, not only the ‘poverty‐hunger‐poor sanitation’ misery triangle, but also access to healthcare, education, energy and heating, etc., particularly in overcrowded environments, such as slums and refugee camps, which to some extent become parallel societies that are very likely to increase in number and scale as mass migrations increase.

## HOW CAN MICROBIAL TECHNOLOGIES CONTRIBUTE TO SOLUTIONS?

While diplomacy plays a central role in the search for peace, attainment of sustainable peace requires that the root causes of conflicts be identified, addressed and resolved. And in this context, microbes have much to offer.

We start with food because, as will be appreciated from regular news bulletins, starvation and malnutrition are ever‐present in many parts of the world.

### Food supply and security

#### Crop yields

One of the most important ecosystem services of microbes is the promotion of plant growth and health by enabling plant access to nutrients, producing biochemicals that inhibit pathogens and by parasitising diverse plant pests (*biopesticides*; Ownley et al., 2004; Pieterse et al., 2014; Roca et al., 2013; Ruffner et al., 2012; Timmis & Ramos, 2021; Trivedi et al., 2017; Verstraete et al., 2022). Moreover, they are key agents of disease‐suppressive soils, soils that contain plant pathogens but whose disease potential is inhibited. The microbes responsible for plant growth promotion are being technically used to increase crop yields and hence food supplies (Bakker & Berendsen, 2022; Hu et al., 2022; Trivedi et al., 2017), and represent *agrobiochemicals* or *agrobiologics*, which can replace costly, energy‐intensive and polluting *agrochemicals* (Braga et al., 2022; Roca et al., 2013; Timmis & Ramos, 2021). In this regard, it should be emphasised that for about a century, increases in crop yields have been to a large extent achieved through the use of relatively inexpensive and amply available fossil fuel‐based nitrogen fertilisers. But these have created a huge environmental burden and have recently become expensive, as a result of soaring energy costs, so urgently need to be replaced by non‐polluting microbial nitrogenase‐based nitrogen – *green nitrogen* (Isobe & Ohte, 2014; Matassa et al., 2023). Agrobiologics can increase local food production and hence contribute to the reduction of human malnutrition, unemployment and poverty (Ramos & Timmis, 2021; Timmis et al., 2017; Timmis & Ramos, 2021).

#### Soil health

Microbes, including the microbial partners of ecosystem engineers such as plants, earthworms and burrowing invertebrates, are key players in soil formation, health and fertility (Blouin et al., 2013; Hullot et al., 2021; Lavelle et al., 2006). (The concept of soil fertility in general assumes a weed‐like crop plant phenotype where the crop grows best in an open habitat, such as loamy soil with high levels of nutrients, good water‐holding capacity, and good drainage (Anderson, 1952). And microbe:plant partnerships are the agents of increases of soil organic carbon, which is a key driver of soil fertility, carbon sequestration and biodiversity (Gougoulias et al., 2014; Sykes et al., 2020), indeed of regenerative agriculture – agricultural practices that improve soil health (Rhodes, 2017). For example, such partnerships in grasslands result in an annual accumulation of about 1 ton of organic carbon per ha, which significantly enhances soil structure, water and nutrient management, and serves to counter climate change (Denman et al., 2007).

#### Marginal soils

Marginal soils are those that, for reasons of aridity, flooding, extremes of temperature, wind erosion, soil structure, poor nutrient content or pollution, are too stressful for plants to grow well and which produce crops whose value barely covers cultivation costs. Global warming and extreme weather events are driving the transformation of productive soils to marginal soils through desertification and erosion of topsoil.

Global warming‐mediated melting of glaciers and the polar ice sheets, and the resulting rise in sea levels, are very much in focus because of displacement of human communities from coastal regions and resulting human migrations. However, another serious consequence is the increase in frequency and extent of inundation by seawater of coastal farmland and its resulting salination, a process that is exacerbated by extreme weather events. Such salination renders fertile agricultural soil inhospitable for most plants and, in turn, also contributes to a reduction in productive farmland, food production capacity and security and, for vulnerable impacted communities, the risk of insufficient nutritional resources.

However, in some cases, the stresses of marginal soils for plants can be alleviated in part by microbial partners, which may either diminish the stressor and/or activate plant metabolic responses that result in elevated levels of plant‐stress tolerance. The harnessing of such microbes to alleviate plant stresses and improve plant growth on marginal soils can extend the range of cultivatable land, increase crop yields and food production, and hence reduce food insecurity in such regions (Alsharif et al., 2020; Egamberdieva et al., 2019; Gao et al., 2022; Maestre et al., 2017).

#### Fermented food and beverages

Microbes provide us with a wide diversity of fermented foodstuffs and beverages. Quite apart from contributing to cultural identity, and new and improved gustatorial and olfactory experiences, fermented foods have reduced perishability and contamination with pathogens and mycotoxins (Adebo et al., 2019). Moreover, fermentation adds to the food material new microbes and new compounds (Kiczorowski, et al., 2022) that may have health benefits, and liberates calories that would otherwise not be accessible to human digestion – this additional energy has helped feed humanity in times of food scarcity since the agricultural revolution. And ensilaging of crops grown for farm animal feed, involving lactic acid bacteria fermentations, also preserves the feed and increases its nutritional value (Guo et al., 2023).

#### Non‐animal husbandry sources of meat

While dietary protein for much of the world's population is provided for via the husbandry of domesticated food animals, other sources of animal meat are exploited, including wild meat (such as bushmeat; see below), insects, etc. Edible insects are generally regarded as a promising sustainable source of protein‐rich food having a much lower climate footprint and agricultural resource consumption footprint (https://www.fao.org/3/i3253e/i3253e.pdf). However, while the risk of zoonotic infections from insects is considered to be less than that from vertebrates, wild insects and farmed insects are known to carry diverse infectious agents that may have potential for transmission to humans (e.g. Gałęcki & Sokół, 2019; Percipalle et al., 2021). It is therefore essential that efforts to promote human consumption of insects include the development of farmed insects with reduced or no associated pathogens, for example by creating germ‐free larvae and populating them with a safe microbiota that provides all essential services for larval growth. Subsequent developments along these lines could be to evolve a microbiota that results in the production of additional/increased quantities of valuable nutrients and even health‐beneficial compounds for the consumer.

#### Microbes as food

Fungi have been a source of food perhaps even from before the times of our hunter–gatherer forebears and, more recently, through commercial mass production of fruiting bodies – the mushrooms – and of mycelium products. However, photosynthetic microbes, like the cyanobacterium *Spirulina/Arthrospira*, which have excellent nutritional composition, promise to become major food sources in the future and facilitate reductions in meat consumption (Garcia et al., 2017). This will have multiple consequences, that are both major in scale and positive in their consequences, because microbial food

- is relatively inexpensive to produce using sunlight and atmospheric CO_2_,
- can be produced locally, reducing costs and creating employment,
- reduces the unnecessary environmental footprint associated with current levels of animal husbandry, including greenhouse gas production, by shifting the focus away from farm animals as primary sources of protein,
- frees up grazing land for other uses
- frees up land used to produce crops for animal feed: Pikaar et al. (2018) calculated that by substituting farmed crops with microbial protein in animal feed, and thus decoupling land us from livestock on a world‐scale basis, an agricultural surface of the size of China could become available for other uses, including nature restoration.

### Sanitation and hygiene

#### Wastewater treatment

Microbes are the great transformers and recycle most of the waste materials produced by organisms of the biosphere, including humans. This natural activity is our most powerful system of sanitation and is technically exploited worldwide to treat human wastes in solid and wastewater treatment plants, which also serve to remove pathogens excreted in our wastes, thereby reducing disease transmission (https://asm.org/Articles/2020/April/How‐Microbes‐Help‐Us‐Reclaim‐Our‐Wastewater).

While the primary need in human societies with insufficient WASH (drinking water, sanitation and hygiene; https://www.who.int/publications/i/item/9789240065031) is the destruction of pathogens, current technologies increasingly focus on the recovery and recycling of the various components of wastes to produce valuable biogas (a mixture of methane and other hydrocarbon gases produced by anaerobic digestion of organic waste materials), compost and re‐usable water (Nielsen, 2017). Thus, the introduction of modern wastewater treatment (WWT) systems in such communities will also extract these valuable resources for local use.

Moreover, WWT will in some cases provide crucial irrigation water for agriculture in regions where this is the rate‐limiting parameter in agricultural productivity.

#### Clean water

Microbes naturally clean water – reducing faecal pathogen and industrial and agricultural pollutant loads – as it percolates through soil and sediments on its way to underlying aquifers that may serve as sources of drinking water. This natural activity is technically exploited worldwide to purify drinking water supplies and safeguard their quality (Favere et al., 2021; Fowler & Smets, 2017; Timmis et al., 2022). And for water supplies that are contaminated with toxic organic pollutants and/or heavy metals, there are promising emerging technologies, such as the use of melanin to bind and filter out heavy metals (Thaira et al., 2019).

Human communities that are located in deserts and coastal regions may have limited access to fresh water. Desalination of seawater or other saline waters is an obvious solution in some instances, but traditional reverse osmosis technology is energy‐intensive and expensive, so is unaffordable by many communities (Xu et al., 2022). Microbial desalination cells couple wastewater treatment to desalination and thus solve two problems simultaneously.

### Healthcare

#### Access to primary healthcare

Disadvantaged (and frequently remote) communities often have a high incidence of diseases, particularly infectious diseases. At the same time, they have poor access to healthcare due inter alia to insufficient funds and infrastructure. There is a humanitarian imperative to provide access to healthcare and medicines to communities that lack them. In addition, infectious diseases that are otherwise treatable and containable can spread, mutate and become uncontainable, epidemic and even pandemic. While there are numerous agencies and philanthropic organisations that provide funds and medicines to impoverished communities, our focus here is the potential of microbial technologies to contribute to solutions.

One concept proposed to improve access to primary healthcare is the creation of modular and independent Do‐It‐Yourself digital medical centres (dmcs; Timmis & Timmis, 2017), which are local and can be readily accessed (24/7). The dmcs are health professional (clinician/pharmacist/advanced practice nurse)‐supervised centres focused (initially) on simple diagnostic tests and basic routine health services that can be carried out by patients themselves, with supervision/assistance by healthcare professionals when required. The DIY dmcs would exploit a growing range of point‐of‐care‐testing kits, such as lateral flow and liquid biopsy tests, and provide corresponding decision tree patient assessment approaches and algorithm/artificial intelligence support for test result analysis, interpretation and recommendation. Such centres would also function for simple non‐DIY healthcare services, such as vaccination campaigns, the provision of tele‐health care (https://catalyst.nejm.org/doi/full/10.1056/CAT.18.0268), and condition self‐management support (https://www.hse.ie/eng/health/hl/selfmanagement/; https://www.hug.ch/sites/interhug/files/structures/endocrinologie_diabetologie_hypertension_et_nutrition/article_who_tpe_eng_slama_golay.pdf). Naturally, a logical extension could include the provision and integration of mobile and wearable community/individual health monitoring and small/digital sample diagnostic devices, bearing various stakeholder needs in mind (Coert et al., 2021). As they would grow in number, dmcs would exert a ‘pull’ for the development of an expanding spectrum of simple diagnostic tests and frugal medical instruments (Ramadurai & Bhatia, 2019; Tran & Ravaud, 2016; https://theconversation.com/frugal‐design‐brings‐medical‐innovations‐to‐communities‐that‐lack‐resources‐during‐the‐pandemic‐147896). A significant fraction of the tests would involve microbial products and activities, such as reporter systems, recombinant antibodies and antigens. Such DIY centres are ideally suited to enable greater access to primary healthcare in both urban and rural settings, and serve to raise awareness of and increase knowledge about personal health. With appropriate investment and capacity building, some of the tests used in the centres could be manufactured locally, and thereby also provide both employment and income to local communities.

The establishment of dmcs should obviously be integrated into the strategic planning of provision of other deficit‐reducing measures, in order to optimise access and delivery of energy requirements. For example, locating dmcs, WWT and drinking water provision facilities together at an accessible location for the community to be served would enable realisation of important synergies and exchanges of services, such as provision of a dmc with energy by an adjacent WWT plant producing biogas; monitoring by a dmc of both drinking water and wastewater streams for pathogens and other community health indicators.

#### Focus on disease prevention

Poverty, hunger and overcrowding all promote disease and – of particular concern – outbreaks and epidemics of infectious diseases. Disease prevention measures, particularly to reduce the transmission of infectious diseases, are vital in communities that do not have modern healthcare systems. Early detection of outbreaks is pivotal to transmission management, and pathogen detection in sewage may alert health authorities earlier than the emergence of the overt disease in the community (Heijnen et al., 2020).

Disease prevention measures also require active participation of community members. Education about disease prevention and transmission of infectious diseases should, therefore, become an integral part of the strategy (see below).

#### Microbiota modulation

Microbes are at the heart of available and in development (neo‐)adjuvant/adjunct therapies, such as pro−/pre−/syn−/post−/pharmacobiotics (Vargason & Anselmo, 2018), faecal microbiota transplantation (El‐Salhy et al., 2021), specific modulation of gut microbiota composition for a variety of physical and mental clinical issues, including uptake and efficacy of oral medicines metabolised by the intestinal microbiota (Clapp et al., 2017), and phages and microbial predators targeting infections by antimicrobial resistant pathogens (Brüssow, 2017a, 2017b; Gordillo Altamirano & Barr, 2019).

#### Access to vaccines and medicines

People in many parts of the world have limited access to vaccines and medicines. Creating local medicine‐ and vaccine‐production facilities, waiving patent and know‐how issues where necessary, and empowering local personnel and supporting capacity building, would improve such situations enormously, as well as locally providing employment, reducing poverty, and improving the local economy (Timmis et al., 2017). In some cases (i.e. in tropical and sub‐tropical regions), it would also help address challenges pertinent to cold chain requirements for unstable medicines. In any case, new medicines and vaccines that do not require cold chains are desperately needed to enable such communities to benefit from adequate prophylactic and therapeutic interventions, so research and development programmes to create these are needed, preferably through the development of HICs – Low‐ and Medium Income Countries (LMICs) partnerships involving newly created research and development facilities in target population countries.

#### Discovery–creation of new vaccines and medicines

Microbes provide or inspire the components of vaccines against infectious diseases, and thus play a key role in disease prevention, especially during crisis situations when devastating outbreaks such as the COVID‐19 pandemic occur (Brüssow, 2021, 2022; Maeda et al., 2021).

Moreover, as a result of their exceptional metabolic diversity, microbes produce an exceptional diversity of chemicals: over 80% of the new compounds discovered that eventually become the structural inspirations for the design of new medicines, including antibiotics, originate in microbes. The microbial world still constitutes a largely unexplored reservoir of new chemicals with different applications awaiting discovery. Many remote communities are located in regions of rich, but under‐explored microbial diversity, which may be expected to yield important new discoveries. Establishment of research and development facilities in such regions would enable exploration and prospecting for new pharmaceuticals (Timmis et al., 2014), with the potential for future commercial development and associated societal and financial rewards, as well as providing new avenues for employment (Timmis et al., 2017) and technology transfer.

### Pollution mitigation

Many impoverished communities suffer from historical or ongoing industrial pollution with toxic chemicals that negatively impact the health of humans, their animals and/or plants and natural ecosystems (Okereafor et al., 2020). Moreover, there can be transmission, and in some cases bioaccumulation, of toxic chemicals within the food chain/food web (https://www.epa.gov/salish‐sea/toxics‐food‐web), all the way to humans. For example:

- fungi accumulate heavy metals – including radioactive metals – so, if grown in contaminated soils/materials and gathered/harvested for human consumption, they can transmit toxic metals to the humans who eat them
- rice accumulates high levels of arsenic
- fish accumulate toxic polychlorinated‐biphenyls and ‐dioxins and humans eat the fish
- *per*‐ and polyfluorinated alkyl substances (PFAS) – ‘Forever Chemicals’ – are a class of more than 4700 synthetic, highly persistent chemicals that do not occur in nature, some of which are known to have adverse health effects and bioaccumulate in the food web (https://chemtrust.org/pfas/#:~:text=PFAS%20(Per%2D%20and%20polyfluorinated%20alkyl,do'not%20occur%20in%20nature; https://www.cdc.gov/biomonitoring/PFAS_FactSheet.html; Lewis et al., 2022; Pickard et al., 2022).

Microbes and/or microbial products (Tran‐Ly et al., 2020), can metabolise, bind and sequester (Cordero et al., 2017; Kumar et al., 2022), detoxify or mineralise pollutants (Boll et al., 2020; Qin et al., 2020; Varjani & Patel, 2017), once the appropriate bioremediation technology is implemented (Wagner‐Döbler et al., 2000). In some instances, plant:microbe partnerships constitute powerful phytoremediation agents (Das, 2018; Kumar et al., 2022; Yang et al., 2020). Bioremediation not only reduces pollutant‐associated health risks but also improves environmental health, which, in turn, counters biodiversity losses. But microbes are not all‐powerful and some pollutants, as exemplified by the super‐recalcitrant PFASs, which are found in all natural environments worldwide (Arslan & El‐Din, 2021). This dramatically illustrates the danger of developing and deploying new synthetic chemicals without knowing whether or not they can be degraded and recycled by microbes at rates that prevent accumulation in the environment, that is without adequate appreciation of relevant biological potentials that will naturally prevent major problems of environmental health with uncertain ecological outcomes.

### Energy and heating

Microbes can generate biofuels through the efficient conversion of agricultural, domestic and industrial wastes to biogas, ethanol and other higher alcohols and biodiesel (Love, 2022; Ramos et al., 2022). Biofuels can reduce fossil fuel dependencies, increase energy security and contribute to an overall decrease in greenhouse gas emissions (Field et al., 2020; Valdivia et al., 2016). And, at higher latitudes, where insufficient energy for heating is problematic, new approaches involving melanin heat‐capture systems offer promise (Cordero et al., 2018).

### Reducing living space, mass migrations and overcrowding

Global warming is melting the polar ice caps and glaciers, causing rises in sea levels that are triggering/will trigger the migration of human communities from coastal to inland regions. In many geographical locations, however, global warming is also reducing habitable space inland by increasing temperatures beyond that which is consistent with habitation, and/or reducing by desertification and soil erosion land productivity below that needed to support current communities that depend on it, again causing migration of peoples to other regions. Other causes of human migrations include insufficient water for drinking, irrigation, domestic use and industrial use; hunger; poverty; overcrowding; disease; discrimination; persecution; and warfare.

Human migrations and displacements result in societies being increasingly squeezed into ever‐tighter habitats, often (though not always) resulting in overcrowding, often associated with significant deficits of resources and services essential to human welfare and, in worst case scenarios, human survival itself (e.g. see Calhoun, 1973), exemplified by the sometimes calamitous conditions that exist in some informal settlements, including refugee camps and slums.

Migrations and overcrowding can create extreme stress for the migrated community, but in some cases also for the original residents of the location involved, and hence tensions that can trigger conflicts (Calhoun, 1973). It is in everyone's best interest to retain as much currently available living space as possible, and reduce physical and cultural displacement, so all ways and means of countering loss of living space and its sustenance potential, including microbial technologies to reduce greenhouse gas emissions, increase plant tolerance of aridity and salinity to increase crop yields and the acreage of productive farmland, and increase water availability (Table 1), must be embraced.

### Poverty–employment–income

Many of the microbial technology interventions discussed above to reduce individual deficits and resource asymmetries, and including education, technology transfer and capacity building activities, will create local employment and, in some cases, generate revenue surpluses. Local employment can, in turn, reduce poverty, which, in of itself, is prime driver of multiple deficits. But longer‐term strategies for sustained improvement should include the establishment of sustainable and integrated socio‐economic programmes that are tailored to the specific resources and opportunities afforded by local conditions. These might include natural product discovery pipelines in regions of high or unique biodiversity (see Discovery–creation of new vaccines and medicines above; Timmis et al., 2014), sustainable biomining of natural resources (Jerez, 2017), and so forth.

This list of microbial technologies having potential to reduce deficits in resources and services (Table 1) is not, of course, comprehensive and there are a number of others that are relevant, including diverse applications of microbial melanins, such as their ability to function as sunscreens (Oh et al., 2021; see also Suryawanshi et al., 2015 for details about prodigiosin as a sunscreen component), capture energy (Cordero et al., 2018), protect against radiation (Cordero et al., 2017; Dadachova et al., 2008) and act as antioxidants, etc. (Roy & Rhim, 2022).

**TABLE 1:** Conflict‐relevant resource deficits and pertinent microbial alleviation technologies^a^

Conflict‐relevant resource deficit	Problems requiring solution	Available microbial technology solutions	Technology solutions under development
Hunger	Suboptimal or unreliable crop yields	Plant growth‐promoting inocula; microbial pesticides; use of manures and other organic materials to boost soil microbes that improve soil structure and fertility WWT to provide irrigation water	
	Marginal and other suboptimal soils	Plant‐stress‐relieving microbial inocula; soil health‐promoting microbes WWT to provide irrigation water	
	Polluted soils	Bioremediation	
	Poor nutrition^b^	Fermented foods Protein‐/vitamin‐rich microbial food	Gut microbiome supplementation Protein‐/vitamin‐rich microbial food in development
	Poor nutrition and disease of food animals	Ensilaging, fermentation of animal food sources Protein‐/vitamin‐rich microbial food Food animal probiotics Vaccines: see *Section Human health* below	Microbiome optimisation for health and yield Protein‐/vitamin‐rich microbial food in development
	Fresh food spoilage/Food wastage^c^	Microbial fermentation to create new, more durable foods (or energy, e.g. biogas) Composting; creation of fertiliser	Microbiological monitoring Use of natural antimicrobials
	Wild animal consumption, exposure to zoonoses	Replacement with alternatives, including microbial food	Creation of local facilities for microbial food production
WASH: Waste and pollution	Human wastes	WWT; composting	
	Industrial pollution	WWT; bioremediation: pollution prevention, treatment at source, e.g. installation of pollutant degradation filters for waste gases	Microbial‐based technologies for a circular economy
	Clinical/ pharmaceutical waste	WWT	Advanced treatment systems with specialist microbes
Drinking water
	General impurities	Sand filters	
	Toxic metals/metalloids	Metal/loid conversion/sequestration systems	Mycofiltration
	Salinity	Microbial desalination cells	2‐G desalination cells
Biofilms	Anti‐biofilm biomolecules	Disintegration of biofilms by phages
Human health	Insufficient access to healthcare		Local DIY digital medical centres

Dearth of affordable medicines/vaccines^e^	Transfer of medicine/vaccine production technology and expertise and creation of local manufacturing facilities^f^	Development of a facilitating patent policy landscape

Many medicines need cold chains in warm climates	Establishment of local medicine production facilities	Creation of stable medicines not requiring cold chains

Transmissible diseases	Provision of vaccines, probiotics	Microbiome interventions to boost immune systems, for prophylaxis, therapy

Water‐borne diseases	Water treatment *(Section WASH, above)*	

Food‐borne diseases	Sanitation *(Section WASH, above)*	

Vector‐borne diseases	Technologies to reduce pathogen transmission, e.g. based on *Wolbachia* and entomopathogenic fungi, such as *Metarhizium*	

Poor nutrition‐/stress‐mediated lowering of disease resilience	*See Sections above to reduce poor nutrition, hunger stress, pollution stress. See section below to reduce poverty stress*	

Sunlight‐related cancers	Sunscreens based on microbial metabolites, such as fungal‐produced melanins and bacterial prodigiosin	
Energy	Insufficient, unreliable or unaffordable energy	Systems for biofuel production from wastes and/or renewables	Bioelectrical systems 2‐G technologies for production of a range of biofuels
Poverty	Lack of employment opportunities	Creation of locally applicable microbial technology‐based enterprises^d^	Investment in pre‐commercial microbial R&D Assessment of local microbial resources for commercialisation
	Under‐exploitation and monetisation of natural resources	Better exploitation, e.g. harvesting and sale of local materials for traditional medicines, licensing of local microbial diversity resources for prospecting for new medicines, biotechnology applications, new foods and food additives, and other purposes	
Reducing living space, mass migrations and overcrowding	Global warming and climate change	Increasing replacement of high carbon footprint animal food by low carbon footprint microbial foods Carbon emission reduction technologies Carbon immobilisation technologies using plant:microbe partnerships	Microbe‐mediated carbon‐capture technologies Climate control technologies, e.g. use of ice‐nucleation bacteria to promote cloud/rain formation
	Food insecurity and reducing productive agricultural land	Plant‐stress‐relieving inocula; soil health‐promoting microbes Use of manures and other organic materials to boost soil microbes that improve soil structure and fertility WWT to provide irrigation water Creation of local facilities for production of microbial food	
	Insufficient clean water	*See Section Drinking water, above*	*See above*
	Poverty	*See Section Poverty, above*	*See above*
Other natural resources	Lack of materials, lack of diversity of materials	Planting trees with microbial inoculants that promote growth and improvement of soil quality Microbial polymer (e.g. cellulose) production technology Microbial fermentations to diversify resources	Restoration of marginal and industrial brownfield soils
	Loss of biodiversity	Production of microbial food to reduce the use of polluting agrochemicals Use of agrobiochemicals (agrobiologics) to replace polluting agrochemicals Use of microbial technologies to reduce deforestation and loss of habitats (biofuels vs. wood as a fuel; microbial inoculants to improve soil fertility and reduce the need for more agricultural land, etc)	
Knowledge, technical expertise, and stewardship of available resources	Insufficient basic knowledge on: ○ impact of microbial activities on wellbeing, e.g. sanitation, the nutritional value of microbial foods ○ available microbial technologies ○ impact of microbial activities on stewardship of resources	Education: training of teachers in societally relevant microbiology and its introduction into school curricula ○ Education and training in regionally relevant microbial technologies ○ Education and training in regionally relevant microbial technologies for resource conservation and sustainable exploitation	Exploration of microbial solutions to specific local problems

Abbreviation: WWT, wastewater treatment.

^a^ See also https://sfamjournals.onlinelibrary.wiley.com/toc/17517915/2017/10/5.

^b^ https://www.fao.org/state‐of‐food‐security‐nutrition/2021/en/.

^c^ https://www.stopfoodlosswaste.org/issue.

^d^ E.g. see Timmis et al. (2014).

^e^ Of course, there are many programmes financing the provision of medicines to LMICs, but here we focus on microbial solutions to deficits.

^f^ A serious barrier to this is current patent practice and policy, which urgently needs addressing in the context of LMICs. Brackets indicate non‐microbial issues that are germane to microbial solution.

## THREE OTHER ISSUES WARRANTING CONSIDERATION

### The consumption of wild meat and issues of zoonotic transmission, epidemics and pandemics

Many animal species consume ‘wild meat’: it is nature in action – predation – a key element of the food chain/web, and was a major source of food for our hunter–gatherer forebears. Today, wild meat may be consumed by humans for diverse reasons, including poverty, recreational hunting, opportunistic situations such as the consumption of ‘road kill’, cultural preferences for wild meat and tradition and culinary appreciation, etc. The presence of wet markets in China and Southeast Asian countries indicates that bushmeat may also be considered as a luxury food item or having medicinal value. This results in significant demand for bushmeat for traditional reasons, but also by affluent individuals in urban areas who appreciate more exotic foods. Indeed, the consumption of bushmeat is part of the culture/religion of diverse communities worldwide, including those of indigenous peoples. And the distinction between wild and domesticated can become blurred with the husbandry of animals like rabbit, deer, boar, ostrich, buffalo, etc.

While the consumption of wild meat has different values for different cultures, our focus is hunger and the consequences of eating wild meat by impoverished communities whose survival depends upon it. The scale of wild meat consumption is significant. One report suggests that it accounts for about 80% of the protein intake of peoples in Central Africa (https://www2.cifor.org/bushmeat), which is comparable to the amount of beef production of Brazil. Other, unique dietary habits can be observed in populations of different indigenous peoples. For example, one marginalised community in India, the Musahars with a population of 2.5 million in the Bihar region, are known as ‘rat eaters’.

Problematically, wild meat is known to be a significant source of infectious agents that are either human pathogens, or can mutate to become human pathogens. For example, bushmeat in Tanzania has been reported to have a high prevalence of *Bacillus anthracis*, *Brucella* and *Coxiella* (Katani et al., 2019, 2021). The risk of zoonotic pathogen transmission associated with meat processing and consumption, which includes Ebola virus, Monkeypox virus, SARS‐CoV and even human immunodeficiency virus (HIV), is very real. The processing of wild meat is a particular risk because of the possibility of direct inoculation of meat‐associated pathogens into cuts and injuries of the handling humans. Wolfe et al. (2005) reported the emergence of zoonotic pathogens in the Cameroon–Congo Basin region between 1970–2005 that included Arboviruses, Ebola, Monkeypox, HIV‐1 and ‐2, Anthrax, Herpes B virus, Simian foamy and other viruses. It has been estimated that up to 10 distinct events of HIV‐1/2 transmission into human populations occurred in the century prior to the global emergence of HIV (Hahn et al., 2000). Infection from the Simian foamy viruses is frequent among hunters in the Republic of Cameroon (Wolfe et al., 2004).

Thus, the consumption of wild meat as a necessity imposed by basic nutritional needs is accompanied by a heightened risk of zoonotic infections and an evolution of animal pathogens/commensals into human pathogens, transmission and the possibility of outbreaks of epidemics/pandemics. Communities dependent on wild meat consumption are in the front line of zoonotic transmissions, which is another element of social inequity, and their nutritional needs may constitute a driver of regional epidemics and potential global pandemics such as COVID‐19.

There is therefore a compelling case for reducing the consumption of wild meat. Key to this will be adequate provision of alternative sources of food that are outlined above. However, traditional, religious and cultural norms will have to be sensitively taken into consideration, and where appropriate accompanied by supporting education and information programmes in relevant topics when offering alternative forms of nutrition.

### Extraction and export of valuable resources with externalisation of pollution cost burdens

There are many examples of regions rich in valuable natural resources, for example gold and other metals, various minerals and petroleum oil, that are exported, but extracted by local workers who see little of the significant economic benefits of the exported resource. Moreover, in many cases, the extraction process creates local environmental pollution that often results in environmental degradation, which is prejudicial to maintenance of biodiversity and ecosystem services (https://www.cbd.int/article/cop15‐cbd‐press‐release‐final‐19dec2022) and, in many cases, with serious adverse effects on the health of the community, on occasion becoming a serious cause of ill‐heath. Regrettably, such pollution is frequently not remediated or the remediation costs are externalised (i.e. the problem is not considered to be the responsibility of relevant transacting partners – the producers and customers) (https://www.imf.org/en/Publications/fandd/issues/Series/Back‐to‐Basics/Externalities; ostrich https://wiki.p2pfoundation.net/Externalization_of_Costs;). Polluting industries that externalise costs are not restricted to the mining of natural resources, and include for example the clothing‐fashion industry. The practice of externalisation of costs is globally pervasive, but has a much greater impact in LMICs because affected communities have little or no power over corporations, criminals, or government actors exploiting their resources.

But pollution creates an important and widespread deficit, that of a clean and healthy environment. Legacy polluted environments must be repaired and future industrial operations changed to prevent pollution. There are powerful microbially based bioremediation technologies that can contribute to the restoration of environmental health and promote biodiversity and the ecosystem services that it provides. There are also effective microbial technologies, for example waste‐gas treatment systems, that can be used to prevent or reduce pollution at source (Komang Ralebitso‐Senior et al., 2012). Furthermore, there exist microbial technologies that enable environmentally friendly extraction processes (Jerez, 2017).

However, the root cause of these problems is the practice of cost externalisation and can only be corrected by a change in political will to require cost internalisation (https://wiki.p2pfoundation.net/Externalization_of_Costs) and proper respect of affected communities and their due rights.

### Microbial behaviour is instructive for us

Microbes also experience deficits and engage in warfare, alliances and cooperation, so it is interesting to consider what we may learn from them in the context of this discussion.

Microbial ecology is a story of constant struggle for survival, a matter of competition for limited habitat space and resources, of pioneer species and predator species, but also a matter of give and take, and – metaphorically speaking – a case of ‘market economy’ at the level of micro‐ and macro‐ecosystems. Recent insights in microbial population dynamics and ecology inform us that cooperation and moderation are the best strategies for long‐term well‐being (Tasoff et al., 2015). Yet, this does not mean ‘equality’ for all at all times: microbial societies may have Pareto distributions in which, for a stable set of conditions, 80% of the resources and the flow of energy appear to be captured by some 20% top performers (e.g. Pelz et al., 1999). While this is also true of some human resources, as proposed by Pareto (https://en.wikipedia.org/wiki/Pareto_principle), and indeed may be a not uncommon aspect of nature, this is not to suggest that such a distribution is a goal to which we should aspire. In any case, any significant change of conditions may result in a new Pareto distribution, that is equilibrium (Marzorati et al., 2008).

On the other hand, drastic perturbation of specific populations or groups of populations of the microbial world, for example though use of pesticides or antibiotics, results in the emergence of certain strains having resistance traits, strains that can be or become disruptive and dangerous (Cray et al., 2013; Flandroy et al., 2018; Jain et al., 2021). Microbial community behaviour clearly shows that such ‘pestering’ should be avoided because it can lead to both weaponising (e.g. proliferation of antimicrobial resistance) and war (e.g. increases in dangerous infections) by the disturbed communities. This commonly occurs in disturbed habitats or other types of open habitats (Cray et al., 2013) and is analogous to power vacuums in human societies, which, historically, also tend to be filled by exploitative, disruptive and dangerous individuals and groups thereof. There exist many (recent) examples of this on various governance levels in all human societies and subsets thereof. This teaches us that change/behavioural modification should be achieved by more clever and balanced strategies.

On the positive side, we have become aware that communication within the microbial world is of crucial importance and underpins the structure and functioning of successful microbial communities (Hughes & Sperandio, 2008).

And, although we may learn much from observing the behaviour of microbial communities, we also need to take into account that microbes also influence our own behaviour directly. Our gut microbiota and the metabolites they create from the food we ingest profoundly influence our mental state, mood and behaviour via the gut:brain axis and the endocrine system (Clarke et al., 2014; Margolis et al., 2021; Smith & Wissel, 2019; https://www.leuvenmindgate.be/news/gut‐bacteria‐may‐influence‐your‐mental‐health). And the nature of the food we eat profoundly influences the composition of our gut microbiota, and hence its influence on our mental state. In other words, thinking about peace at the level of human society needs to take into account the fact that human attitudes may to some extent be influenced by the microbial constitutions of people's guts, which, in turn, are influenced by the food they eat and, importantly, the foods they do not eat/cannot obtain.

The ability to study the behaviour of millions of microbes at the single cell level, and the ecosystem services they provide, can yield important insights into microbial ecology (Wood, 2022) that, in constellations when interaction is primarily transactional, can help guide the design of certain parts of human ecosystems.

## SO WHY AREN'T MICROBIAL TECHNOLOGIES BEING EFFECTIVELY EXPLOITED WHERE THEY ARE MOST NEEDED?

Besides political will – a proper discussion of which is beyond the scope of this contribution – one of the hindrances to effective and timely exploitation of microbial technologies that can contribute to attainment of the SDGs and improvement of the human condition is the general lack of knowledge about microbes, what they do, and how they have been and can be harnessed to carry out processes that are transformational for humanity (exemplified by vaccines, wastewater treatment). One example of this neglect is the fact that microbes are seldom considered in the context of strategies to mitigate climate change, despite being responsible for the generation and consumption of some powerful greenhouse gases such as carbon dioxide, methane and nitrous oxide (Cavicchioli et al., 2019; Sykes et al., 2020; Tiedje et al., 2022). In the minds of most people, microbes are to be feared, because ‘they’ are invisible actors that can have serious adverse effects by producing diseases like meningitis and pandemics such as acquired immune‐deficiency syndrome (AIDS) and COVID‐19, or cause souring of the milk/moulding of the bread and other forms of food deterioration, sometimes producing food poisoning, or red tides that ruin our fish industries and prevent us from swimming. But these ‘baddies’ are the exception and most microbes are either harmless, helpful or essential to our well‐being and that of the entire biosphere. Most of the bioeconomy is based on microbial activities. Even the ‘baddies’ can have a positive side: for example, the viruses that cause hepatitis have also provided the means to prevent hepatitis by enabling the creation of highly effective vaccines; tetanus toxin that kills us enables us to create inactive tetanus toxoid vaccine that protects us from disease. And battles with pathogens over human evolutionary time have sharpened our immune system via natural selection (Rook, 2022).

So we must:

- urgently counter germophobia and help establish a more balanced and, above all, better informed perception of microbes, in order that society can form realistic, more granular opinions of microbes, their benefits and dangers (Finlay & Arrieta, 2016; Gupta et al., 2020; Timmis et al., 2019),
- convince the public and policy‐makers that the entire biosphere, and the multitude of ecosystem services it provides and upon which we depend, is sustained by the Earth's microbiome,
- reveal how the microbiomes of individuals provide vital services to all organisms, including humans, without which we would not be able to survive for any period of time.
- emphasise that microbial technologies, such as vaccine and antibiotic production, wastewater treatment and sanitation, clean drinking water provision, and fermented foods, have more than any other societal development transformationally improved life expectancy and quality for much of humanity,
- convey how microbial technologies, such as amino acid and vitamin production, bioremediation, seed inoculants, biocatalysis, etc., contribute greatly to human well‐being and endeavours
- explain that microbes are at the centre of biogeochemical cycles that on one hand, provide us with oxygen to breathe and, on the other, play crucial roles in many of the crises facing humanity, like global warming (Cavicchioli et al., 2019), eutrophication‐oxygen minimum zones‐loss of biodiversity, desertification, etc., and hence must receive due consideration in efforts to confront these crises, and, above all,
- convince society to see microbes as solutions not just problems, and to vigorously explore microbial activities for new solutions to existing and anticipated crises.

All of this underscores the vital need for improved knowledge on the causes of and potential solutions to deficits, on sustainability, ecosystem services and their interconnected nature and impact on human and biosphere well‐being. For society to optimally take advantage of the products and services that microbes can deliver to improve societal well‐being, reduce inequalities and level up, and reduce conflicts, people must become microbiology literate (Timmis et al., 2019). Knowledge of societally relevant microbiology must be democratised and provided to all humans, albeit in forms that are catered to their needs. To quote Louis Pasteur: ‘“Science knows no country, because knowledge belongs to humanity, and is the torch which illuminates the world’.

The International Microbiology Literacy Initiative (IMiLI) is creating teaching resources that will form comprehensive curricula of societally‐relevant microbiology that can be integrated into the school curricula of all countries with education authorities willing to embrace them. These resources are being created pro bono by hundreds of microbiologists worldwide and will soon be made freely available. They not only provide relevant knowledge about microbial activities that touch our lives and those of other members of the biosphere (including those of the atmosphere: see Šantl‐Temkiv, et al., 2022), but also explain their relevance within the context of the SDGs, and so contribute to teaching about sustainability in schools and colleges, and their relevance to decision‐making and stakeholder scrutiny of decisions, and so will teach about stewardship and stakeholder responsibilities. Most importantly, these resources aim to expose the wealth of activities of microbes that can be harnessed for the good of humanity, including the promotion of peace in the world.

And, in the context of decision‐making, it is worth remembering that our gut microbes digest much of our food intake and convert it to a form we can use and, in so doing, create a plethora of metabolites, some of which are essential for us, and some of which affect our moods and, in turn, influence our decisions (Smith & Wissel, 2019). Without them, we would indeed be highly stressed: they are personal peace‐makers for each of us.

## REDUCING DEFICITS IN BASIC RESOURCES REALISES SYNERGIES IN SOCIETAL BENEFITS

Humanity has a responsibility to ensure that all peoples of the world have access to resources fundamental to a basic quality of life, or if necessary provide them. This is essential to sustainable development of the human race, and peace and harmonious co‐existence of nations, and is eloquently formulated in the SDGs elaborated by the United Nations. Microbial activities are relevant to most, some would argue all, of the SDGs.

But here we focus on those that can specifically contribute to peace in the world, inter alia the provision of adequate food, sanitation and clean water, the production and supply of antibiotics and vaccines, especially during the appearance of devastating outbreaks, improving soil health, plant health and crop yields, and efforts to slow loss of habitable land by reducing global warming. All these measures are microbe‐centric, as are a range of other relevant technologies (Table 1). Investing in these technologies in regions of the world where they are needed should bring exceptional fruits in terms of elevating the human condition from unacceptable to acceptable and, ideally, beyond just reducing inequalities and promoting peace.

And, it should be pointed out that the technologies outlined above should in some instances also be more generally exploited to increase resource security worldwide, especially but not only in times of crisis (Timmis et al., 2022).

We started out by emphasising the polydeficit nature of impoverished communities, the fact that such deficits are interdependent and mutually re‐enforcing in nature. However, this interconnectedness is not only a characteristic of problems but also of solutions: introducing new technologies to solve problems such as hunger, poor sanitation, access to vaccines and medicines, etc., also creates synergies and facilitates demographic change and dividends by shifting the focus from risk reduction (and survival) towards mid‐ and long‐term (individual to national) planning based on improved health, education, employment (Timmis et al., 2017) and productivity gains. This, therefore, elevates well‐being on a number of fronts. Lifting fellow humans out of impoverishment not only advances the levelling‐up process and gain of dignity but promotes harmony among peoples and removes some root causes of conflict.

Let us break the vicious circle of deficits and misery that promote conflicts, and conflicts that promote deficits and misery. Let us hinder weaponisation of deficits. Let us weaponise microbial technologies to reduce resource deficits and promote resource symmetries among nations, to reduce conflicts, and to promote societal equity, harmony and stability and cooperation between nations. Let us weaponise microbial technologies for peace!

## RECOMMENDED ACTIONS TO IMPLEMENT RELEVANT MICROBIAL TECHNOLOGY SOLUTIONS TO DEFICITS

We need to urgently supply to communities lacking adequate levels of basic resources/services the infrastructure and know‐how (capacity building), and funding for

1. use of agrobiologics to increase crop yields, by providing green nitrogen, stimulating plant growth, and combatting pathogens and pests,
2. exploitation of plant:microbe partnerships to improve soil health and implement regenerative agriculture,
3. creation of nutritious fermented food from locally available crops,
4. better use of microbes in the feed and food supply chains,
5. production of microbial food for humans and farm animals,
6. drinking water production and quality safeguarding,
7. waste treatment with resource recovery,
8. creation of modular DIY digital medical centres,
9. production of vaccines and medicines,
10. bioremediation and biorestoration of the environment in general and natural ecosystems in particular, to create healthier habitats and promote biodiversity (see also: https://www.cbd.int/article/cop15‐cbd‐press‐release‐final‐19dec2022),
11. reduction of greenhouse gas production and capturing carbon,
12. production of biofuels,
13. creation of local employment opportunities associated with the above,
14. development of transdisciplinary approaches, using chemistry‐related (Kondratov et al., 2022), computation technologies (Schultes et al., 2019), psychology‐related (Vinuesa et al., 2020) and other approaches that are synergistic to microbial solutions and
15. education in societally relevant microbiology

## FINAL REMARK

In this Editorial, we draw the reader's attention to various challenges and potential microbial biotechnology‐based solutions (or those inspired by microbiology) that could address and reduce inequalities across the globe, which are chiefly driven by (in many cases meanwhile unnecessary) resource asymmetries that can lead to human suffering and desperation. We do not claim to be complete or prescriptive on how the thoughts and suggestions we have collated here affect, apply to or adequately reflect specific unmet needs and priorities of an overwhelmingly diverse set – across the globe – of inter alia different peoples, cultures and traditions, local and regional circumstances, interdependencies and forms of governance and policies. And we are aware that viewpoints and approaches suggested here might be adequate for one setting but not another (apparently appropriate) setting. Careful and culturally sensitive exploration and appraisal of potential solutions is of paramount importance when considering any changes to the *status quo* and thus must be tightly coordinated and validated with, and driven by (pull), stakeholders for which pertinent approaches are supposed to provide specific improvements. They should not be driven primarily by what facilitators (donors/funders, policy‐makers, implementing organisations, etc.) believe to be adequate or of utility; naturally, expert consultation with and by facilitators notwithstanding.

Our aim is to inspire readers and diverse stakeholders to critically consider the different points we raise, the potential solutions we suggest and the notions and implications we provide, and, if appropriate, advocate for pertinent appraisal within their specific target contexts and application settings of unmet need. We also hope that this contribution will further inspire innovators (and give them confidence in their efforts) to focus on inventing and developing technologies that can help reduce resource asymmetries and inequalities – and promote peace – across the world.

## AUTHOR CONTRIBUTIONS

**Kenneth Timmis:** Conceptualization (equal); writing – original draft (equal); writing – review and editing (equal). **Shailly Anand:** Writing – review and editing (equal). **John E. Hallsworth:** Writing – review and editing (equal). **James Timmis:** Writing – review and editing (equal). **Willy Verstraete:** Writing – review and editing (equal). **Arturo Casadevall:** Writing – review and editing (equal). **Utkarsh Sood:** Writing – review and editing (equal). **Roshan Kumar:** Writing – review and editing (equal). **Princy Hira:** Writing – review and editing (equal). **Charu Dogra Rawat:** Writing – review and editing (equal). **Abhilash Kumar:** Writing – review and editing (equal). **Sukanya Lal:** Writing – review and editing (equal). **Rup Lal:** Writing – review and editing (equal). **Juan Luis Ramos:** Writing – review and editing (equal).

## FUNDING INFORMATION

Natural History Museum; Indian National Science Academy.

## CONFLICT OF INTEREST Statement

The authors confirm they have no conflicts of interest.
